# A Comprehensive Review of Recent Advances in Minimally Invasive Glaucoma Surgery: Current Trends and Future Directions

**DOI:** 10.7759/cureus.65236

**Published:** 2024-07-24

**Authors:** Kasturi K Dhawale, Pravin Tidake

**Affiliations:** 1 Ophthalmology, Jawaharlal Nehru Medical College, Datta Meghe Institute of Higher Education and Research, Wardha, IND

**Keywords:** ophthalmic surgery, clinical outcomes, surgical innovations, glaucoma management, intraocular pressure (iop), minimally invasive glaucoma surgery (migs)

## Abstract

Glaucoma, a leading cause of blindness globally, necessitates effective management strategies to prevent irreversible vision loss. Traditional glaucoma surgeries, while effective, are associated with significant risks and complications. Minimally invasive glaucoma surgery (MIGS) has emerged as a transformative approach, offering safer and less invasive alternatives. This review provides a comprehensive overview of recent advancements in MIGS, highlighting current trends, technological innovations, and future directions. MIGS procedures, characterized by smaller incisions and quicker recovery times, have expanded the therapeutic landscape, enabling earlier intervention and improved patient outcomes. The review evaluates various MIGS techniques, their efficacy, safety profiles, and clinical outcomes, drawing insights from comparative studies and meta-analyses. Technological innovations, including enhanced device designs and integration with digital health technologies, have further bolstered the field. Despite challenges in patient selection and long-term outcomes, the future of MIGS is promising, with ongoing research and development poised to enhance its impact. By synthesizing the latest research, this review aims to inform clinicians, researchers, and policymakers, ultimately contributing to improved management of glaucoma and patient care.

## Introduction and background

Glaucoma is a group of eye conditions that damage the optic nerve, often due to abnormally high intraocular pressure (IOP). It is one of the leading causes of blindness worldwide, affecting over 70 million people [1]. Glaucoma is often called the "silent thief of sight" because it typically progresses without noticeable symptoms until significant vision loss has occurred. The primary types of glaucoma are open-angle glaucoma, the most common form, and angle-closure glaucoma, which is less common but can cause sudden vision loss. Effective management of glaucoma is crucial to prevent irreversible blindness, and this requires a combination of early detection, regular monitoring, and appropriate treatment [2].

Minimally invasive glaucoma surgery (MIGS) represents a paradigm shift in the surgical management of glaucoma. Traditional glaucoma surgeries, such as trabeculectomy and tube shunt implantation, are highly effective but come with significant risks and potential complications. MIGS procedures aim to reduce IOP with a lower risk profile, making them suitable for earlier intervention in the disease course [3]. Smaller surgical incisions, reduced tissue manipulation, and quicker recovery times characterize these procedures. MIGS has gained popularity due to its ability to bridge the gap between medication therapy and more invasive surgical options, providing a safer and more effective alternative for many glaucoma patients [4].

This review aims to provide a comprehensive overview of the recent advances in minimally invasive glaucoma surgery. It aims to highlight the current trends in MIGS, evaluate the efficacy and safety of various MIGS procedures, and discuss technological innovations and future directions in this rapidly evolving field. By synthesizing the latest research and clinical outcomes, this review offers valuable insights for clinicians, researchers, and policymakers involved in managing glaucoma, ultimately contributing to improved patient care and outcomes.

## Review

Current trends in MIGS

Types of MIGS Procedures

**Figure 1:**
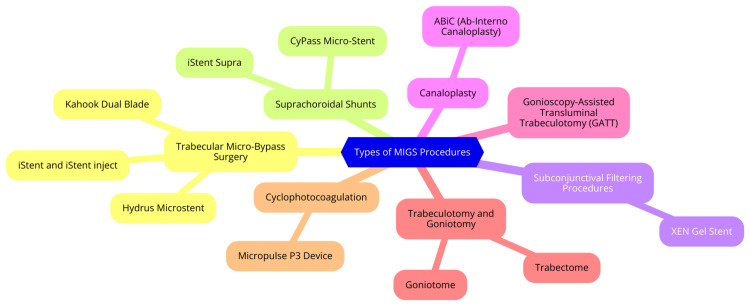
Types of minimally invasive glaucoma surgery (MIGS) procedures Image Credit: Dr. Kasturi K. Dhawale

Minimally invasive glaucoma surgery (MIGS) procedures can be categorized into four main types based on their mechanism of action and surgical approach. Trabecular meshwork stents enhance aqueous outflow through the conventional pathway by bypassing the trabecular meshwork. Examples include the iStent, iStent Inject, and Hydrus Microstent, typically implanted during cataract surgery and suitable for mild to moderate glaucoma [3]. Suprachoroidal shunts create a pathway between the anterior chamber and the suprachoroidal space, increasing uveoscleral outflow. The CyPass Micro-Stent (withdrawn from the market) and iStent Supra are examples [3]. Subconjunctival devices create an alternative outflow pathway into the subconjunctival or sub-Tenon's space, bypassing the conventional system. The Xen Gel Stent is used for mild to severe glaucoma [5]. Cycloablative procedures decrease aqueous production by targeting the ciliary body. Endocyclophotocoagulation (ECP) and micropulse cyclophotocoagulation are examples used in various glaucoma stages. The selection of a MIGS procedure depends on factors such as glaucoma type, severity, patient characteristics, surgeon expertise, and desired IOP reduction. Trabecular meshwork stents are preferred for mild to moderate glaucoma, while subconjunctival devices and cycloablative procedures are used in advanced cases [6]. Types of MIGS procedures are shown in Figure 1.

Efficacy and Safety Profiles

The efficacy and safety profiles of minimally invasive glaucoma surgery (MIGS) devices vary depending on their mechanism of action and targeted outflow pathway. Generally, MIGS devices offer a more favorable safety profile than traditional glaucoma surgeries, with reduced risks of complications such as infection, vision loss, and hypotony [7,8]. Devices targeting Schlemm's canal, like the iStent and Hydrus Microstent, exhibit the most favorable safety outcomes but typically provide a moderate reduction in intraocular pressure (IOP) [7]. Studies indicate similar efficacy in IOP reduction between the iStent inject and Hydrus, with the potential for reduced medication use with the iStent inject [9]. However, the Hydrus may carry a higher risk of complications and subsequent procedures than the iStent injection [9]. Suprachoroidal devices, exemplified by the CyPass Micro-Stent, offer the potential for greater IOP reduction but may be associated with unpredictable IOP spikes and hypotony [7]. Subconjunctival devices, such as the Xen Gel Stent, can achieve significant IOP reduction but may fail due to subconjunctival fibrosis or result in complications related to bleb formation [7]. The proportion of annual iStent procedures has notably increased in recent years, surpassing traditional glaucoma surgeries by 2016 [9], indicating a growing preference for MIGS devices among surgeons and patients. Choosing the appropriate MIGS device should be guided by considerations such as the severity of the patient's glaucoma, the desired level of IOP reduction, and tolerance for potential complications. While MIGS devices represent a safer alternative to conventional glaucoma surgeries, ongoing research, and long-term studies are essential to further assess their efficacy and safety across diverse patient populations and disease severities.

Comparative Studies and Meta-Analyses

Minimally invasive glaucoma surgery (MIGS) devices yield important insights into their clinical utility. Lavia et al. (2017) conducted a systematic review and meta-analysis comparing MIGS techniques, either standalone or combined with cataract surgery, against medical therapy, cataract surgery alone, other glaucoma surgeries, and alternative MIGS procedures. They found that MIGS devices significantly lowered intraocular pressure (IOP) and reduced the number of glaucoma medications at 12 months compared to medical therapy. However, they emphasized the necessity for more high-quality studies to substantiate these findings [10]. Mathew and Buys (2020) critically evaluated the MIGS literature and highlighted a substantial proportion of trials that do not adhere to World Glaucoma Association (WGA) guidelines. This lack of adherence limits inter-study comparisons and hinders a comprehensive evaluation of MIGS technologies. They advocated adherence to research publication's WGA guidelines to enhance transparency and facilitate meaningful comparisons between studies [11]. Davids et al. (2020) assessed the 12-month outcomes of ab interno canaloplasty (ABiC), a novel MIGS procedure. Their study reported significant reductions in IOP and the number of glaucoma medications, accompanied by a favorable safety profile. Nonetheless, they underscored the necessity for larger, longer-term studies to validate these initial findings [3]. While MIGS have demonstrated promise in lowering IOP and decreasing reliance on glaucoma medications, the quality of evidence varies among studies. There is a pressing need for additional high-quality, independently funded comparative studies to comprehensively evaluate MIGS devices' efficacy, safety, and cost-effectiveness across varying degrees of glaucoma severity. Adherence to standardized guidelines, particularly those advocated by the WGA, is crucial for improving study quality and enabling robust comparisons across different MIGS interventions.

Technological advances

Innovations in Device Design

Minimally invasive glaucoma surgery (MIGS) has seen remarkable advancements in device design, driving its evolution and broader adoption in glaucoma management. These innovations have notably enhanced the efficacy, safety, and applicability of MIGS procedures. One pivotal area of innovation involves the development of next-generation stents and devices that target different pathways of aqueous humor outflow. These advancements include refined trabecular bypass technology and novel approaches to enhancing uveoscleral and subconjunctival outflow. Examples like the iStent, Hydrus microstent, and others are tailored to specific aspects of aqueous humor dynamics, offering targeted solutions [12]. A significant breakthrough is the emergence of drug-eluting MIGS devices capable of performing surgical interventions while delivering medications directly to target tissues. This dual functionality optimizes intraocular pressure (IOP) control while reducing reliance on topical medications. MIGS devices are engineered to be biocompatible, durable, and supportive of natural aqueous outflow, ensuring sustained efficacy and safety over the long term [3]. Innovative patented designs, such as the multidirectional stent of devices like the iStent Infinite, bypass trabecular resistance while preserving most of the trabecular meshwork. This precision allows for more targeted interventions and restores physiological outflow dynamics effectively [13]. Moreover, advancements in injector systems, exemplified by those used with the iStent Infinite, offer unlimited stent delivery attempts, boosting surgeon confidence and procedural ease regardless of experience level. These improvements in injector design enhance the accessibility and reliability of MIGS procedures [13]. These innovations in MIGS device design have significantly broadened these interventions' procedural capabilities and safety profiles. By targeting diverse anatomical pathways and integrating advanced materials and technologies, MIGS devices are increasingly versatile, providing tailored solutions for a wide spectrum of glaucoma patients [13].

Improved Surgical Techniques

Minimally invasive glaucoma surgery (MIGS) has seen significant advancements recently, driven by efforts to enhance surgical techniques for improved efficacy, safety, and usability. These advancements aim to address the challenges of existing MIGS procedures and deliver better outcomes, particularly for patients with mild to moderate glaucoma. Key improvements include refinements to established techniques like the Trabectome and iStent inject procedures to enhance their effectiveness and simplify their application. Combination MIGS procedures, which involve employing multiple MIGS devices or techniques during a single surgery, have also been explored to amplify the efficacy of intraocular pressure (IOP) reduction [3]. The OMNI Surgical System exemplifies this trend by integrating two MIGS procedures in one operation: creating a new internal channel for fluid shunting and enlarging the natural drainage pathway to Schlemm's canal. Several new MIGS devices have been developed to overcome the limitations of existing models, introduce novel mechanisms for aqueous outflow, improve user-friendliness, or enhance device efficacy. These innovations include the MINIject DO627 by iStar Medical, the Intra-Scleral Ciliary Sulcus Suprachoroidal Microtube, the iDose TR by Glaukos Corporation, the Beacon Aqueous Microshunt by MicroOptx, the Minimally Invasive Micro Sclerostomy (MIMS) from Sanoculis Ltd., and the STREAMLINE® Surgical System by New World Medical. These MIGS surgical technique advancements promise to improve patient outcomes through more effective IOP reduction, reduced complication risks, and enhanced ease of use for surgeons. Continued research and development are expected to yield further enhancements in MIGS techniques and devices, expanding the options available for managing glaucoma [14].

Advances in Imaging and Diagnostics

Advances in medical imaging and diagnostics have transformed healthcare by enabling more precise diagnoses, personalized treatment strategies, and improved patient outcomes. Key innovations include the integration of artificial intelligence (AI) and machine learning, which analyze extensive imaging datasets to assist radiologists in detecting abnormalities, enhancing diagnostic accuracy, and reducing interpretation time. AI also supports risk assessment and facilitates personalized treatment planning. Three-dimensional (3D) imaging and reconstruction techniques, such as computed tomography (CT) and magnetic resonance imaging (MRI), provide detailed anatomical views that enhance visualization, aid in surgical planning, and enable targeted interventions. Functional imaging modalities like functional MRI (fMRI) and positron emission tomography (PET) capture metabolic and functional data, enabling clinicians to assess brain activity, identify organ function irregularities, and monitor treatment responses in conditions like cancer and neurological disorders [15]. Hybrid imaging technologies, such as PET-CT and single-photon emission computed tomography (SPECT)-CT, combine modalities to offer comprehensive diagnostic information in a single examination. This approach provides precise localization of abnormalities, facilitating accurate diagnosis and treatment planning. Advancements in imaging also support image-guided interventions, enabling minimally invasive procedures with enhanced precision, reduced risks, and quicker recovery times [16]. Portable ultrasound devices deliver real-time imaging at the bedside, aiding in rapid diagnosis, continuous monitoring, and guiding interventions. Further innovations include the development of contrast agents and radiopharmaceuticals, which improve diagnostic accuracy by enhancing imaging quality. Nearly 60% of ongoing developments in diagnostic imaging involve these compounds [17]. Integrating AI, advanced imaging agents, and ultrasound technology will streamline testing processes, boost diagnostic accuracy, and expand diagnostic capabilities in 2024. This convergence of technologies promises to enhance the efficiency, accuracy, and accessibility of diagnostic tests, thereby playing a pivotal role in preventive healthcare and early disease detection [18].

Integration With Digital Health Technologies

Minimally invasive glaucoma surgery (MIGS) has seen remarkable technological advancements in recent years, driving its evolution and broadening its application in managing glaucoma. These innovations have notably enhanced the efficacy, safety, and versatility of MIGS procedures. Key technological advancements in MIGS include developing micro-surgical equipment and techniques to minimize tissue trauma and accelerate postoperative recovery. Specialized micro-surgical instruments enable precise interventions with minimal surgical impact [19]. Advanced microscopes and intraoperative imaging technologies, such as high-definition gonioscopy and anterior segment optical coherence tomography (OCT), enhance angle structure visualization. This aids in precise device placement and enhances procedural safety [19]. Significant progress has been made in the design and materials of stents and scaffolds used in MIGS. These devices are engineered to be biocompatible, durable, and supportive of natural aqueous outflow. Examples such as the iStent, Hydrus microstent, Trabectome, and Kahook Dual Blade (KDB) address specific aspects of aqueous humor dynamics, contributing to effective IOP management [20]. Integrating advanced diagnostic tools like OCT into preoperative planning enhances procedural precision and customization in MIGS. Real-time monitoring technologies are critical during surgery because they enable immediate adjustments and ensure procedural accuracy and safety. These technological advancements have significantly expanded MIGS's capabilities and safety profile, allowing surgeons to perform more precise and minimally invasive procedures. Improving surgical techniques has led to better patient outcomes, reduced surgical risks, and faster recovery times, making MIGS an increasingly attractive option for a wider range of glaucoma patients. The field of MIGS continues to evolve rapidly, with ongoing research and development focusing on new technologies and techniques. A notable example is the FDA approval of the STREAMLINE Surgical System in October 2021, underscoring the ongoing innovation in this field [21].

Clinical outcomes

Patient Selection Criteria

When surgeons select patients for minimally invasive glaucoma surgery (MIGS), several critical factors come into play. The severity of glaucoma is a primary consideration, with MIGS typically recommended for patients with mild to moderate glaucoma who are often on one or two medications. Patients with severe glaucoma or advanced visual field loss may benefit more from invasive procedures like subconjunctival filtration, which offer greater intraocular pressure (IOP) reduction [3]. Assessment of angle anatomy is crucial, as MIGS is most suitable for both primary and secondary open-angle glaucoma. A detailed evaluation using gonioscopy helps identify landmarks and assess the feasibility of canal-based procedures. Combined MIGS and cataract surgery benefit patients with both conditions, synergizing IOP reduction [22]. The surgeon's expertise with a specific MIGS procedure is vital for patient selection, particularly in managing complex cases. Starting with simpler cases and gradually gaining experience is recommended. Patients must have realistic expectations regarding achievable IOP and medication reduction with MIGS. Individuals willing to undergo multiple procedures and prioritize vision preservation are ideal candidates for minimally invasive options, while those needing more significant IOP reduction may require more invasive approaches [22]. Additionally, ocular and systemic factors such as inflammation, uveitis, or the use of anticoagulants should be considered for their potential impact on surgical outcomes. Selecting patients for MIGS involves comprehensively evaluating glaucoma severity, angle anatomy, cataract status, surgeon expertise, patient expectations, and relevant ocular and systemic factors. Thoughtful patient selection is crucial for optimizing outcomes and minimizing complications [22].

Short-Term and Long-Term Outcomes

Recent studies have shown promising outcomes for minimally invasive glaucoma surgery (MIGS) procedures when combined with cataract surgery, both in the short-term (≤12 months) and long-term (>12 months). In short-term analyses, a retrospective study comparing phacoemulsification combined with iStent and VISCO360 versus phaco-iStent alone demonstrated greater mean intraocular pressure (IOP) reduction (2.9 ± 3.6 mmHg vs. 1.7 ± 3.1 mmHg) and a higher proportion achieving IOP <18 mmHg with the same or fewer medications (46% vs. 35%) at six months [23]. Another retrospective study examining 12-month outcomes of phaco combined with iStent and endocyclophotocoagulation (ICE) versus phaco-iStent in mild to severe open-angle glaucoma (OAG) found the ICE group achieved greater mean IOP reduction (7.14 vs. 4.48 mmHg) and medication reduction (63% vs. 38%) [23]. Additionally, a study evaluating the OMNI surgical system as a standalone procedure in pseudophakic eyes reported a 69.8% success rate at six months based on criteria including IOP reduction and medication stability [23]. In long-term assessments (>12 months), a study analyzing 12- to 18-month outcomes of various MIGS procedures (iStent, Hydrus, and Xen) combined with cataract surgery revealed a mean IOP decrease of 2.2 mmHg, a reduction of 1.2 glaucoma medications, and an improvement in visual acuity by 0.13 logMAR. Visual field metrics remained generally stable, with the XEN stent group showing the most significant decrease in glaucoma medications compared to iStent and Hydrus at follow-up [23]. Another study of the OMNI system as a standalone procedure in mild to moderate OAG reported an 8.0 mmHg reduction in IOP from a baseline of 26.6 mmHg at 24 months, with patients using an average of 1.9 fewer medications [24]. These findings underscore the effectiveness of MIGS procedures combined with cataract surgery in achieving sustained reductions in IOP and medication use, enhancing visual outcomes, and maintaining stable visual fields over short and long-term periods.

Quality of Life Assessments

Minimally invasive glaucoma surgery (MIGS) has revolutionized glaucoma treatment by offering a less invasive alternative to traditional surgeries, aiming to reduce intraocular pressure (IOP) with minimal tissue trauma and enhanced safety profiles. These procedures are categorized into angle-based (e.g., Trabectome, iStent), suprachoroidal (e.g., CyPass Micro-Stent), and subconjunctival (e.g., Xen Gel Stent) MIGS, each targeting specific sites for implantation or augmentation. Recent advancements include surgical technique refinements and the introduction of innovative devices like the FDA-approved STREAMLINE Surgical System. Despite their advantages, challenges remain in efficacy for severe cases, long-term data availability, and accessibility. Ongoing research and technological innovations continuously improve MIGS capabilities and expand their applications, promising further advancements in glaucoma management and patient outcomes [12]. Clinical studies have demonstrated promising outcomes for MIGS procedures combined with cataract surgery. For instance, a retrospective study comparing phacoemulsification combined with iStent and VISCO360 versus phaco-iStent showed superior mean IOP reduction and a higher proportion of achieving target IOP levels with fewer medications at six months [25]. Another study comparing phaco combined with iStent and endocyclophotocoagulation (ICE) versus phaco-iStent in mild-severe open-angle glaucoma reported greater reductions in both IOP and medication use in the ICE group [25]. In longer-term analyses (12-18 months), studies of MIGS devices (iStent, Hydrus, and Xen) with cataract surgery revealed significant IOP reductions, decreased medication dependency, and improvements in visual acuity while maintaining stable visual field metrics. The XEN stent particularly demonstrated substantial decreases in glaucoma medication use compared to other devices [25]. Assessing the impact of MIGS on quality of life (QoL) has been crucial. Post hoc analysis of the iStent inject trial indicated improved QoL measures for patients undergoing combined MIGS/cataract surgery versus cataract surgery alone [26]. A prospective comparison between MIGS (iStent, trabectome) and trabeculectomy found no significant differences in QoL parameters at six months post-surgery [26]. The development of tools like the Glaucoma Outcomes Survey (GOS) addresses specific QoL concerns such as functional limitations, discomfort from eye drops, and psychosocial impacts identified through patient focus groups. Despite the documented benefits of reducing IOP and medication burden, further large-scale studies using standardized QoL assessments are needed to comprehensively evaluate patient-reported outcomes with MIGS procedures [26].

Future directions

Emerging Technologies in MIGS

Emerging technologies are driving significant advancements in minimally invasive glaucoma surgery (MIGS), ushering in new possibilities for treating glaucoma more effectively. One notable area of innovation involves artificial intelligence (AI) and machine learning technologies, such as Eyenuk, which are being explored to enhance early screening and diagnosis of glaucoma. This early detection could facilitate timely interventions and personalized treatment plans, potentially improving patient outcomes [27]. Another pivotal development lies in the creation of novel MIGS devices. Companies focus on specialized markets and disruptive technologies to innovate and challenge established norms. For example, the FDA-approved STREAMLINE Surgical System (New World Medical) features a single-use, disposable device designed with a stainless-steel cutting inner cannula and a polymer outer sleeve, offering a new approach to glaucoma management [28]. Advancements in suprachoroidal MIGS are also noteworthy, aiming to enhance uveoscleral outflow through innovative devices like the iStent Supra and CyPass. Similarly, in subconjunctival MIGS, novel devices such as the EX-PRESS Glaucoma Filtration Device, Xen Gel Stent, and PreserFlo MicroShunt are creating alternative aqueous outflow pathways, expanding treatment options for clinicians, and improving patient outcomes [29]. Collaborative efforts are pivotal for the success of these emerging technologies. MIGS companies actively support global fellowship programs and training missions to educate surgeons in developing nations. This collaboration among industry, healthcare providers, and policymakers is essential for enhancing the accessibility and sustainability of these innovative technologies worldwide [30]. As these emerging technologies continue to evolve and gain acceptance, they hold tremendous promise for transforming glaucoma management, reducing disease burden, and broadening access to advanced MIGS procedures on a global scale. The future of MIGS appears bright, poised to revolutionize the approach to glaucoma care and elevate standards of patient treatment worldwide [12].

Potential for Combination Therapies

Combination therapies have emerged as a promising strategy for managing glaucoma, offering enhanced efficacy in lowering intraocular pressure (IOP) and improved patient convenience compared to monotherapy. Fixed-combination medications have garnered significant attention due to their distinct advantages [31]. Examples such as Cosopt (dorzolamide/timolol), Combigan (brimonidine/timolol), and Simbrinza (brinzolamide/brimonidine) have demonstrated superior IOP-lowering effects compared to their components alone. These medications streamline the dosing regimen, reducing the number of daily eye drops and exposure to preservatives, which can enhance patient adherence and persistence with treatment. In the United States, three fixed-combination medications are FDA-approved: Cosopt (dorzolamide/timolol), Combigan (brimonidine/timolol), and Simbrinza (brinzolamide/brimonidine). Similar fixed combinations are available in various countries worldwide [32]. Fixed combinations achieve IOP reduction through different mechanisms, such as decreasing aqueous humor production (dorzolamide, brinzolamide, timolol) and enhancing outflow (brimonidine, prostaglandin analogs). Researchers are actively investigating novel fixed combinations, including prostaglandin analogs combined with other classes of medications, to further improve IOP-lowering efficacy and enhance tolerability [33]. These ongoing efforts aim to expand treatment options for glaucoma patients, potentially leading to more effective management strategies with reduced treatment burdens.

Personalized Medicine Approaches

Personalized medicine, or precision medicine, represents a cutting-edge approach to disease prevention and treatment that tailors interventions to individual patients based on their unique genetic profile, environment, and lifestyle [34]. This approach aims to deliver precise treatments to patients at the right time, offering several advantages over traditional, one-size-fits-all medicine. Key elements of personalized medicine include genetic profiling, targeted therapies, early intervention, and improved patient outcomes. Advances in next-generation sequencing (NGS) technologies have enabled rapid identification of genetic variations in individuals, facilitating more accurate diagnoses and personalized treatment plans. By understanding a patient's genetic makeup, personalized medicine allows for the development of more effective therapies that are less likely to cause adverse effects than conventional treatments. Genetic testing can also reveal predispositions to specific diseases, enabling early detection and proactive preventive measures before symptoms appear. Ultimately, personalized medicine aims to achieve more precise diagnoses, earlier interventions, and more efficient drug development processes, improving patient outcomes and potentially reducing healthcare costs [35]. However, widespread adoption faces challenges such as the high costs associated with molecular profiling tests, the need for specialized knowledge and computational expertise to interpret genetic data, and the necessity for streamlined methods to integrate personalized healthcare approaches into existing healthcare systems. Despite these challenges, ongoing research and technological advancements continue to refine the capabilities of personalized medicine. The field is evolving rapidly, with ongoing efforts to overcome barriers and realize its full potential to transform healthcare delivery and enhance patient care [36].

Challenges and Limitations

Minimally invasive glaucoma surgery (MIGS) has transformed the landscape of glaucoma treatment, yet it grapples with several challenges and constraints. Primarily indicated for patients with mild-to-moderate glaucoma, MIGS devices often demonstrate limited efficacy compared to traditional surgical options in advanced cases. Furthermore, the available long-term data on their safety and effectiveness remain sparse, necessitating ongoing research and clinical observation to establish comprehensive outcomes over extended periods [12]. The accessibility of MIGS procedures is also hindered by their cost and availability, particularly in resource-limited settings. Efforts are crucial to enhance affordability and broaden access to these technologies. Moreover, mastering MIGS techniques demands specialized training and experience. Surgeons transitioning from traditional glaucoma surgeries may encounter a steep learning curve, emphasizing the importance of structured training programs to facilitate proficiency [37]. Certain MIGS devices have encountered specific challenges. For instance, the CyPass Micro-Stent was withdrawn from the market due to the significant endothelial cell loss associated with its use. Careful patient selection and meticulous device positioning are imperative to mitigate such risks. Similarly, suprachoroidal MIGS devices, while capable of substantial IOP reduction, pose concerns regarding unpredictable efficacy, postoperative hypotony, and sudden IOP spikes [38]. Despite these obstacles, ongoing research and technological advancements continue to refine MIGS capabilities and expand their clinical applications. As surgeons accumulate experience and a broader array of MIGS approaches emerge, these procedures hold potential for broader utilization in more severe glaucoma cases and across diverse clinical scenarios [12].

## Conclusions

MIGS has revolutionized the landscape of glaucoma treatment, offering a promising alternative to traditional surgical approaches. This review highlights the significant advancements and trends in MIGS, emphasizing its efficacy, safety, and potential for earlier intervention. Technological innovations, such as improved device design and integration with digital health technologies, have further enhanced the effectiveness of MIGS procedures. While challenges remain, particularly regarding patient selection and long-term outcomes, the future of MIGS is bright with ongoing research and development. As the field continues to evolve, MIGS holds the potential to significantly improve the quality of life for glaucoma patients by providing safer, less invasive, and more effective treatment options. Clinicians, researchers, and policymakers must stay abreast of these advancements to ensure optimal patient care and address the growing global glaucoma burden.
